# Oxidative Stress Status in COVID-19 Patients Hospitalized in Intensive Care Unit for Severe Pneumonia. A Pilot Study

**DOI:** 10.3390/antiox10020257

**Published:** 2021-02-07

**Authors:** Joël Pincemail, Etienne Cavalier, Corinne Charlier, Jean-Paul Cheramy–Bien, Eric Brevers, Audrey Courtois, Marjorie Fadeur, Smail Meziane, Caroline Le Goff, Benoît Misset, Adelin Albert, Jean-Olivier Defraigne, Anne-Françoise Rousseau

**Affiliations:** 1 Clinical Chemistry, CHU of Liège, Sart Tilman, 4000 Liège, Belgium; etienne.cavalier@chuliege.be (E.C.); e.brevers@chu.ulg.ac.be (E.B.); c.legoff@chu.ulg.ac.be (C.L.G.); 2 Toxicology Department, CHU of Liège, Sart Tilman, 4000 Liège, Belgium; c.charlier@chuliege.be; 3 Department of Cardiovascular Surgery, CHU of Liège, Sart Tilman, 4000 Liège, Belgium; JP.Cheramy@chuliege.be (J.-P.C.-B.); a.courtois@chuliege.be (A.C.); jo.defraigne@chuliege.be (J.-O.D.); 4 Service of Diabetology, Nutrition and Metabolic Diseases, CHU of Liège, Sart Tilman, 4000 Liège, Belgium; marjorie.fadeur@chuliege.be; 5 Institut Européen des Antioxydants, 54000 Nancy, France; smeziane@ie-antioxydants.com; 6 Intensive Care Department, CHU of Liège, Sart Tilman, 4000 Liège, Belgium; benoit.misset@chuliege.be (B.M.); afrousseau@chuliege.be (A.-F.R.); 7 Biostatistics and Medico-economic Information Department, CHU of Liège, Sart Tilman, 4000 Liège, Belgium; aalbert@uliege.be

**Keywords:** COVID-19, oxidative stress, critical care, vitamin C, lipid peroxides

## Abstract

Background: A key role of oxidative stress has been highlighted in the pathogenesis of COVID-19. However, little has been said about oxidative stress status (OSS) of COVID-19 patients hospitalized in intensive care unit (ICU). Material and Methods: Biomarkers of the systemic OSS included antioxidants (9 assays), trace elements (3 assays), inflammation markers (4 assays) and oxidative damage to lipids (3 assays). Results: Blood samples were drawn after 9 (7–11) and 41 (39–43) days of ICU stay, respectively in 3 and 6 patients. Vitamin C, thiol proteins, reduced glutathione, γ-tocopherol, β-carotene and PAOT^®^ score were significantly decreased compared to laboratory reference values. Selenium concentration was at the limit of the lower reference value. By contrast, the copper/zinc ratio (as a source of oxidative stress) was higher than reference values in 55% of patients while copper was significantly correlated with lipid peroxides (r = 0.95, *p* < 0.001). Inflammatory biomarkers (C-reactive protein and myeloperoxidase) were significantly increased when compared to normals. Conclusions: The systemic OSS was strongly altered in critically ill COVID-19 patients as evidenced by increased lipid peroxidation but also by deficits in some antioxidants (vitamin C, glutathione, thiol proteins) and trace elements (selenium).

## 1. Introduction

Severe acute respiratory syndrome coronavirus 2 (SARS-CoV-2) was responsible for Coronavirus disease 2019 (COVID-19), the first cases having been reported in Wuhan, China, in December 2019 [1]. Due to fast transmission and pathogenicity, the coronavirus has spread across all countries, provoking a pandemic [2]. COVID-19 is characterized by aberrant host immune response, leading to excessive inflammatory responses (or cytokine storm)—as evidenced by high blood levels of cytokines, chemokines and C-reactive protein—and is associated with severe damage to the respiratory system and multi-organ failure, contributing to fatal outcomes of infected patients [3,4,5]. Post-mortem analysis of COVID-19 lungs showed infiltration of inflammatory mononuclear cells and macrophages in the air spaces, which may induce diffuse remodeling of the alveolar wall [6].

In a large number of pathologies, inflammation is known to be closely related to oxidative stress, one process being easily induced by the other [7,8,9]. Oxidative stress (OS) is defined as an imbalance between toxic reactive oxygen species (ROS) and antioxidants in favor of oxidants, leading to a disruption of redox signaling and/or irreversible oxidative damage to lipids, deoxyribonucleic acid (DNA) or proteins [10]. Oxidative damages are involved in the development of different pathologies including cancer, cardiovascular, neurodegenerative and lung diseases [11]. Besides inflammation, other pathophysiological mechanisms in relationship with increased OS have been advocated to explain the pathogenesis of COVID-19 [12]: inhibition of angiotensin converting enzyme 2 (ACE-2) activity [13,14,15,16], denaturation of hemoglobin leading to iron metabolism dysregulation with release of toxic free iron ion [17,18,19,20,21], disseminated intravascular coagulation due to hypoxia [12] and endothelial dysfunction [22,23,24,25,26].

Despite recent papers highlighting the key role of oxidative stress and inflammation duo in the development of COVID-19 [12,27,28], little information is available about the systemic oxidative stress status (OSS) of COVID-19 patients hospitalized in intensive care unit (ICU) for severe pneumonia. The present study aimed to specifically identify blood biomarkers of OS including antioxidants, trace elements, and oxidative damages to lipids in such COVID-19 patients and to analyze their relationship with inflammation as a major source of ROS production.

## 2. Material and Methods

This study was conducted during the first wave of the COVID-19 pandemic in May 2020 in the 50-bed Intensive Care Unit (ICU) of the University Hospital of Liège in Belgium. Due to the particular context of the pandemic, we received first a written agreement from the President of local Ethical Committee on 29 April 2020. The study protocol was then formally approved under national reference B707202000035, Local reference 2020-201, on 5 June 2020. All legal representatives of the patients were instructed on the study objectives and signed informed consent.

The study was conducted on a convenience sample of critically ill adult patients hospitalized for severe COVID-19 pneumonia. Patients were excluded if they were weaned from mechanical ventilation and if they were on continuous veno-venous hemofiltration (CVVH) during the 7 days before blood sampling. In total, nine patients were included.

All the patients received medical nutrition following the local nutritional practices. According to the nutritional guidelines that were available at the time of the study, weight-based formulas were used to estimate energy and protein targets (Table 1). Enteral nutrition was administered continuously using a volumetric pump. Gastric residual volume was monitored every 6 h: in case of volume ≥250 mL, feeding rate was reduced and prokinetics (metoclopramide or erythromycin) were considered at the intensivist’s discretion. The different industrial enteral solutions were: Nutrison Protein Plus, Nutrison Energy Multi Fibre, (Nutricia^®^, Brussels, Belgium) and Peptamen AF (Nestlé Health Science^®^, Brussels, Belgium). In case of insufficient or contraindicated enteral nutrition, respectively supplemental or total parenteral nutrition was initiated using a ternary mixture (Smofkabiven, Fresenius Kabi^®^, Schelle, Belgium). Parenteral nutrition was administered continuously via a central venous line, using a volumetric pump. Patients on parenteral nutrition or continuous veno-venous hemofiltration were supplemented with micronutrients (Addaven^®^, Soluvit^®^ Novum, Vitalipid^®^ Novum Adult (Frésénius Kabi^®^, Schelle, Belgium)).

Demographic data (age, sex, weight, height, body mass index (BMI), medical history, severity scores, organ support duration, route of nutrition, length of stay (LOS), survival) were collected for all patients from the electronic medical record.

Blood samples drawn from a venous central line on tubes containing EDTA and citrate were immediately centrifuged at 3000× *g* during 10 min. Serum gel was allowed to clot during 30 min before being centrifuged. Plasma and sera samples were then frozen at −80 °C until analysis of OS biomarkers. Blood determination of antioxidants, respectively vitamins C and E (γ- and α-tocopherols), vitamin E standardized to cholesterol (Vit E/cholesterol), β-carotene, glutathione (GSH), thiol proteins (PSH), glutathione peroxidase (GPx), trace elements (selenium (Se), copper (Cu), zinc (Zn), copper/zinc ratio), biomarkers of oxidative damages to lipids (lipid peroxides (ROOH), oxidized LDL (ox-LDL), and antibodies IgG against oxidized LDL (Ab-ox-LDL) has been described in detail [29,30]. C-reactive protein (CRP) concentration was determined on a COBAS^®^8000 analyzer (Roche Diagnostics, Machelen, Belgium). Myeloperoxidase (MPO) was assessed using MPO ELISA kit (Immun Diagnostik, Bensheim, Germany). Cholesterol was measured using an enzymatic method with cholesterol oxidase on Abbott Alinity C (Abbott, Chicago, IL, USA). Albumin was assessed by spectrophotometry using Alinity C kit (Abbott, Chicago, IL, USA). White blood cells and neutrophils were determined by flow fluorocytometry on Sysmex-SN device (Ontario, Canada). The total antioxidant capacity (TAC) of plasma was evaluated by using the PAOT^®^ (Pouvoir AntiOxydant Total, Institut Européen des Antioxydants, Nancy, France) score as previously described [31]. Briefly, the measurement was carried out in a reaction medium (1 mL physiological solution at pH ranging from 6.7 to 7.2, temperature 24–27 °C) containing a molecule in a free radical state called mediator (M•). Two microelectrodes, one being the working electrode and the second one the reference electrode, were then immersed in the medium. After addition of 20 μL of plasma, the PAOT activity was estimated by registering electrochemical potential modifications in the reaction medium. Blood concentrations of all OS biomarkers were compared to the reference values applicable in the central laboratory of the CHU of Liège [29,30].

Patients were divided in two groups according to the ICU stay duration. Patients with an ICU stay ≤10 days (short stayers, N = 3 patients) or >10 days (long stayers, N = 6 patients) before blood sampling were arbitrarily defined as short or long stayers, respectively.

## 3. Statistical Analysis 

Quantitative data were expressed as median and range while numbers and percent were used for categorical findings. To compare the distribution of biological parameters of COVID-19 patients with the laboratory reference intervals, we used the sign test based on the binomial distribution. Specifically, according to the sign test, when all COVID-19 patient values fell below (or above) the middle of the reference interval, we concluded that the biological test was significantly lowered (or increased) in COVID-19 patients (*p* = 0.004) and likewise for eight COVID-19 patients (*p* = 0.040); otherwise, COVID-19 patients were not statistically different from presumably healthy subjects. Comparison of OSS between short and long stayers was made using the nonparametric Mann-Whitney U test. Results were considered significant at the 5% critical level (*p* < 0.05). The Spearman correlation coefficient was calculated to measure the association between biological parameters. A *p*-value < 0.05 was considered as statistically significant.

## 4. Results

The characteristics of the nine study patients are described in Table 2. Most of the patients were overweight and/or presented pathologies such as type 2 diabetes (64%) or arterial hypertension (66%).

All the patients were on enteral feeding and one patient received supplemental parenteral nutrition during the 7 days before blood sampling. During the entire ICU stay before blood sampling, 3/9 patients received temporary supplemental parenteral nutrition in addition to insufficient enteral nutrition, and 3/9 patients had been temporarily on CVVH. These patients received an intravenous supplementation in vitamins and trace elements. Mean daily macro- and micronutrients intakes from ICU admission to blood sampling are described in Table 3.

As shown in Table 4, the median concentration of vitamin C, γ-tocopherol, β-carotene, PSH, GSH and PAOT^®^ score was statistically lower than the reference interval when considering all patients. By contrast, those of GPx, MPO, neutrophils count and CRP was significantly higher than the standards. Figures S1–S3 (see Supplementary Material) show all the individual OS biomarkers values when compared to the reference interval. Vitamin C, PSH and GSH concentrations were below the lower normal value (LNV) in almost all patients. By contrast, elevated levels in GPX ROOH, copper/zinc ratio and CRP being higher than the upper normal value were found in a large majority of patients.

Table 5 displays the main significant correlations observed between OS biomarkers. Copper correlated positively with ROOH, Cu/Zn ratio and CRP but negatively with γ-tocopherol. Cu/Zn ratio tended to positively correlate with ROOH and similarly for Cu and CRP. By contrast, γ-tocopherol tended to correlate negatively with ROOH, ox-LDL and CRP. MPO negatively correlated with albumin and, to a lesser extent, with PSH. No correlation was found between PSH and albumin (data not shown).

As blood samples were taken after different times in the ICU, we investigated if differences could occur in OS biomarkers concentration between short and long stayers (Table S1, see Supplementary Material). Only a significant increase in vitamin C, β-carotene and selenium was observed in long stayers when compared to short stayers. Nevertheless, the median value of both antioxidants always remained below the INV. By contrast, those of selenium has changed from 51 (28–67) µg/L in short stayers to 97.5 (73–105) µg/L in long stayers, this last value being in the reference interval. GSH concentration decreased in a non-significant way from 794 µM in short stayers to 598 µM in long stayers, a value largely below the INV. As shown in Table S2 (see Supplementary Material), abnormal values in vitamin C, PSH, GSH, GPx, copper/zinc ratio, ROOH, neutrophils and CRP were frequent (≥50%) in both groups. Low selenium levels were mostly observed in short stayers (Figure S2, see Supplementary Material).

## 5. Discussion

To the best of our knowledge, this is the first report showing a deep alteration of systemic OSS using an analysis of a large number of biomarkers in COVID-19 patients hospitalized for severe pneumonia. Of interest to note is that results were homogenous despite a different timing (short and long stayers) in the blood collection.

### 5.1. Antioxidant Analysis

An important collapse in antioxidant defenses was detected in the COVID-19 patients as evidenced by levels of vitamin C, GSH, PSH, γ-tocopherol and β-carotene that were largely below the reference interval.

When considering all the nine COVID-19 patients, their median value in vitamin C corresponds to the definition of a hypovitaminosis C [32]. Despite a significant increase when compared to short stayers, vitamin C level in long stayers remained below the inferior normal value. In a recent paper, Chiscano-Camón et al. [33] reported that undetectable vitamin C levels were observed in 94.4% of COVID-19 patients 17.5 days after ICU hospitalization. In our study, such a vitamin C deficiency indicated that intakes of this antioxidant given at nutritional doses as recommended by ESPEN guidelines [34] were therefore not sufficient to maintain vitamin C concentration in the reference interval, as already observed in other critically ill patients [35]. In fact, intravenous (IV) administration only has been able to restore high-level ascorbic acid plasma concentration [36]. Even if always being a matter of controversy [37,38], high doses (several grams) of IV vitamin C could help reducing the “cytokines storm” [39] and could have immunosuppressive effects [40]. When compared to a placebo group, Zhang et al. [41] recently demonstrated in a preprint paper without peer-reviewing that high-doses of IV vitamin C (12 g every 12 h) for 7 days improved oxygenation in COVID-19 critically ill patients. Moreover, blood interleukin-6 levels were significantly reduced after 7 days of treatment. Unfortunately, the authors did not monitor serum vitamin C concentration before and after IV injection.

Glutathione, a crucial antioxidant, is well known to modulate the behavior of many cells including the cells of the immune system, augmenting the innate and the adaptive immunity as well as conferring protection against microbial, viral and parasitic infections [42,43]. Interaction between GSH metabolism and several diseases were also well described [44]. For example, low glutathione has been associated with abnormalities in the lung surfactant system, while normal levels of intracellular glutathione may exert a critical negative control on the elaboration of pro-inflammatory cytokines [45]. Our study revealed that the GSH status was significantly altered downwards in our COVID-19 patients, particularly in the long stayers. Recently, Polinokov [46] concluded that blood deficiency in GSH exacerbated COVID-19 illness. In two COVID-19 patients, Horowitz et al. [47] observed that the 2 g per os or IV glutathione improved their dyspnea within 1 h of use. Repeated use of 2 g was effective in further relieving respiratory symptoms. Recently, Poe and Corn, Rangel-Mendez et al. and De Flora et al. [48,49,50] hypothesized that N-acetyl-L-cysteine (NAC) as a precursor of glutathione could act as a potential therapeutic agent in the treatment of COVID-19 through a variety of potential mechanisms, including increasing glutathione, improving T cell response, and modulating inflammation. Interestingly, we also observed in parallel a deep depletion in PSH. By its large amount in plasma, albumin dotted of –SH groups represents around 75% of the thiol pool [51]. Infectious and inflammatory states are known to alter the serum concentration of albumin [52], as observed in the present study. Nevertheless, we found no correlation between PSH and albumin.

The determination of the total antioxidant capacity (TAC) has been proposed as a global measure of non-enzymatic antioxidant efficiency despite the problem of inference with uric acid [53,54]. Using an original electrochemical methodology developed by us [31], we showed that the TAC as evaluated by the PAOT^®^ score was logically decreased in COVID-19 patients most probably because of their very low levels in vitamin C and GSH. Nevertheless, no correlation could be evidenced between both antioxidants and TAC as also shown in the EPIC Granada–Gipuzkoa study [55]. Nevertheless, it was interesting to note the positive correlation of PAOT^®^ score and the Vit E/cholesterol. More investigations are required to better understand and evaluate the real role and place of TAC in the OSS.

### 5.2. Trace Elements Analysis

Adequate levels of Se are important for initiating immunity, but they are also involved in regulating excessive immune responses and chronic inflammation [56]. In the present study, selenium was close to the inferior normal value, and an important deficit was detected in the three short stayers. Moghaddam et al. [57] analyzed the selenium status in a cohort of 33 COVID-19 patients providing a set of four consecutive serum samples taken from ICU admission to ICU discharge (median: 19 (3–46) days) or death (median: 10 (2–32) days). When compared to reference data issued from a European cross-sectional analysis, these authors found, in agreement with us, a pronounced deficit with a very low selenium concentration in 43.4% of the samples, more particularly in those of short stayers (Figure S2, see Supplementary Material). By contrast, all our long stayers exhibited individual values being within the reference interval, significantly higher than those of short stayers. This could suggest that the nutritional intakes in selenium could have been adequate to compensate initial deficits.

In their paper, Moghaddam et al. [57] also analyzed glutathione peroxidase (GPx), an antioxidant enzyme requiring glutathione (GSH) and selenium as co-factors to eliminate lipid peroxides (ROOH). Unlike the present results, these authors found a significant correlation between selenium and GPx. Even if the GPx level was elevated in all our patients, it should be surprising that its activity was optimal given the low level in its co-factor GSH but also the elevated concentration in ROOH.

Copper exhibits, at non-physiological concentration, pro-oxidant activities by inducing free radical formation through Fenton-like reaction [58]. The potential pro-oxidant effect of copper was suggested in our study by both its strong positive correlation with ROOH and negative correlation with γ-tocopherol, a major antioxidant acting as lipid peroxidation inhibitor. Besides an important role in immunity [59], zinc also had antioxidant properties [60] as main co-factor of superoxide dismutase (SOD) but also as inhibitor of free radical reaction induced by copper. As shown in Figure S2 (see Supplementary Material), Cu/Zn ratio values higher than the UNV were found in half of the patients. As part of an OS assessment, some authors suggested that the Cu/Zn ratio should be considered as a better indicator of OS presence than copper alone [61,62]. Such a ratio is rarely analyzed in critically ill patients. The strong relationship between health status and Cu/Zn ratio has, however, been extensively reviewed by Malavolta [63]. So, human studies have well evidenced that inflammatory stimuli modify serum concentration of Cu and Zn by increasing the former and decreasing the latter through a hepatic organized mechanism [64]. In our study, we effectively observed that the Cu/Zn ratio was positively correlated with CRP. Moreover, the ratio also tended to be positively correlated with ROOH (Table 5). This is in agreement with previous papers reporting that increased copper/zinc ratio was correlated with heightened systemic oxidant load in aging-related degenerative diseases [65], patients undergoing renal dialysis [66] or women taking oral contraceptive [30].

### 5.3. Analysis of Lipid Oxidation Biomarkers

Even if the elevated level of lipid peroxides was not statistically different from the reference interval, a majority of COVID-19 patients (63.6%) exhibited higher levels than the upper reference value (Figure S2, see Supplementary Material). The strong correlation observed between ROOH and copper highlighted the important role of this last trace element in the development of the lipid peroxidation process. By contrast, ox-LDL, as another form of lipid peroxidation [67], did not confirm the presence of increased oxidative damages to lipids. However, ox-LDL elicited the production of both IgG and IgM ox-LDL antibodies. It is accepted that IgG ox-LDL antibodies had pro-inflammatory effect while IgM were anti-atherogenic [68]. Surprisingly, we observed in our study two distinct profiles in IgG values (Figure S2, see Supplementary Material). We have no clear explanation related to such discrepancy.

### 5.4. Analysis of Inflammation Biomarkers

In COVID-19 patients as well as in critically ill patients, increased plasma inflammatory markers such as CRP have been well described [69]. In our study, we confirmed such inflammatory process (Table 4) as especially evidenced by elevated levels in MPO, an enzyme specifically localized in neutrophils. This reflects the activation of these cells probably by cytokines leading to the release in the extracellular medium of ROS in high amount [70]. MPO can also catalyze the formation of toxic hypochlorous acid (HOCl), able to oxidize albumin [71]. Oxidation of –SH groups of albumin is known to occur in pathophysiological processes associated with increased inflammation and oxidative stress [51] and potentially in COVID-19 patients [72]. This could explain why we have observed a negative correlation between MPO and albumin in our patients.

Our observational study did not allow us to conclude if increased OS and antioxidant depletion could be directly attributed to the COVID-19 disease by itself or by its complications and the delivered organ supports [73,74,75]. Nevertheless, significant alterations in vitamin C, GSH and selenium concentrations, all molecules playing a key role in the immunity, raise questions in the particular framework of the COVID-19 pathogenesis. Associated to increased lipid peroxidation, these observations should therefore create opportunities to explore potential approaches for prevention or treatment by antioxidants, as observed in COVID-19 patients [76,77,78,79,80]. As recently suggested for vitamin C and glutathione [33,47,81], monitoring of OSS should be implemented in COVID-19 critically ill patients, including vitamin C, GSH in association with PSH, and, finally, ROOH in association with the Cu/Zn ratio and CRP.

### 5.5. Study Limitations

First, only a few number of patients have been studied and blood sampling timing was not standardized. Second, some clinical data, such as the number of septic complications, were not taken into account in the present analysis. Moreover, OSS status of our patients at ICU admission was unfortunately unknown. Our patients were overweight (median value: 29 kg/m^2^) and some of them had pre-existing medical conditions such as diabetes (64%) and/or arterial hypertension (55%), all conditions being potentially associated with basal increased OS [82,83,84]. If focusing on vitamin C, the literature review indicated, however, that these pathological situations by themselves could not be responsible for hypovitaminosis C [85,86,87,88]. Finally, nutritional support varied between patients and their average energy and protein intakes was lower than recommended. This could have influenced the observed OS status.

## 6. Conclusions

The systemic OSS was strongly altered in critically ill COVID-19 patients as evidenced by increased lipid peroxidation but also by deficits in some antioxidants (vitamin C, glutathione, thiol proteins) and trace elements (selenium). Strong positive correlations between lipid peroxides and Cu and the negative correlation between γ-tocopherol and Cu highlighted the role played by copper in increased OS in COVID-19 patients. These observations need to be confirmed on a larger population.

## Figures and Tables

**Table 1:** Nutrition calculations. y: years; * BMI: body mass index; CVVH: continuous veno-venous hemofiltration; ** IBW: ideal body weight.

Body Weight Considered for Nutritional Calculations	
Eutrophic patients: * < 75y: BMI 18.5–25 kg/m^2^ * ≥ 75y: BMI 23–28 kg/m^2^	Actual weight: measured in hospital or obtained from patient’s recent medical history
Underweight patients	* IBW = expected weight for * BMI = 18.5 kg/m^2^ if age < 75y * BMI = 23 kg/m^2^ if age ≥ 75y
Overweight patients	IBW = expected weight for * BMI = 25 kg/m^2^ if age < 75y * BMI = 28 kg/m^2^ if age ≥ 75y
Obese patients: * < 75y: BMI ≥ 30 kg/m^2^ * ≥ 75y: BMI ≥ 30 kg/m^2^	Adjusted IBW = ** IBW + 0.25 × (actual weight − IBW)
Nutritional targets	
Eutrophic, underweight and overweight patients	Energy: 25 kcal/kg/d Protein: 1.2 g/kg/d (1.7 g/kg/d if CVVH)
Obese patients	Energy: 20 kcal/kg/d Protein: 2 g/kg/d

**Table 2:** Demographic data of COVID-19 patients (N = 9). BMI: body mass index; LOS: length of stay; SAPS II: simplified acute physiology score II; ICU: intensive care unit. Data are expressed as median (P25-P75) or number (%).

Variable	Summary Statistics
Age (y)	64 (53–71)
Sex ratio (M/F)	8/1
Weight (kg)	90 (81–102)
Height (cm)	173 (169–181)
BMI (kg/m2)	29.4 (28.4–32.3)
Active smoking, n (%)	1 (11)
Active alcoholism, n (%)	1 (11)
Pre-existing medical conditions:	
- Type 2 diabetes, n (%)	6 (66)
- Arterial hypertension, n (%)	6 (66)
- Gastric sleeve surgery	1 (11)
SAPS II	33 (25–45)
ICU LOS (d)	54 (42–65.5)
Hospital LOS (d)	63 (49–91)
Mechanical ventilation duration (d)	38 (20–49)
CVVH during ICU stay, n (%)	1 (11)
Enteral nutrition during ICU stay, n (%)	9 (100)
Supplemental parenteral nutrition during ICU stay, n (%)	3 (33)

**Table 3:** Daily nutritional intakes in vitamins, trace elements and lipids in COVID-19 patients (N = 9) hospitalized in ICU. Data are expressed as median (P25–P75).

Variable	Summary Statistics
Cu (mg/day)	1.4 (1–1.8)
Zn (mg/day)	12.7 (8.6–15.7)
Se (µg/day)	72.3 (46.8–79.9)
Vitamin C (mg/day)	124.2 (94.6–172.1)
Vitamin E (mg/day)	17 (12.6–21.5)
Vitamin A (µg/day)	1012 (712.8–1182)
Lipids (g/day)	42.7 (27.8–54.2)
Energy (kcal/day)	1263 (899.8–1389)
Proteins (g/day)	65.2 (47.6–77.7)

**Table 4:** Statistical comparison of OS biomarkers in all COVID-19 patients (N = 9) with their reference interval using the sign test based on the binomial distribution (see statistical analysis). k: number of COVID-19 values below * or above ** the middle of reference interval.

Variable	Reference Interval	Median (Range)	k	*p* Value
Antioxidants				
vitamin C (µg/mL)	6.21–15.18	3.91 (3.06–6.14)	9 *	0.004
vitamin E as α-tocopherol (µg/mL)	8.60–19.24	17.90 (13.3–21.1)	3 *	1
vitamin E/cholesterol (µg/g)	4.4–7.0	10.92 (9.14–13.16)	0 *	1
α-tocopherol (µg/mL)	0.39–2.42	0.84 (0.57–1.28)	8 *	0.040
β-carotene (mg/L)	0.06–0.68	0.14 (0.11–0.28)	9 *	0.004
thiol proteins (µM)	314–516	250 (204–258)	9 *	0.004
glutathione (µM)	717–1110	629 (508–697)	8 *	0.040
oxidized glutathione (µM)	0.96–10	<0.96	0 **	1
PAOT^®^ score (U/L)	1.46–36.74	10.52 (6.63–10.77)	9 *	0.004
glutathione peroxidase (UI/g Hb)	20–56	69.55 (61.90–78.27)	9 **	0.004
albumin (g/l)	32–46	28 (27.5–33.0)	8 *	0.040
Trace elements				
copper (mg/mL)	0.70–1.10	1.16 (0.66–1.47)	5 **	1
zinc (mg/mL)	0.70–1.20	0.84 (0.81–1.09)	5 *	1
selenium (µg/L)	73–110	74 (59–103)	5 *	1
Biomarkers of lipid peroxidation				
ROOH (µM)	0–432	674 (181–1415)	6 **	0.50
ox-LDL (ng/mL)	28–70	50 (36–70)	5 **	1
Ab-ox-LDL (IU/L)	200–600	306 (64–1200)	4 *	1
Sources of ROS production				
copper/zinc ratio	1.00–1.17	1.55 (0.79–1.69)	5 **	1
white blood cells (10^3^/mm^3^)	4.60–10.10	8.42 (7.07–13.03)	6 **	0.50
neutrophils (%)	42–71	75.6 (60.8–86.3)	8 **	0.04
myeloperoxidase (ng/mL)	27–72	88 (60–191)	8 **	0.04
C-reactive protein (mg/L)	0–5	32.8 (9.6–59.8)	8 **	0.04

**Table 5:** Correlation between some OS biomarkers as observed in COVID-19 patients. ROOH: lipid peroxides, CRP: C-reactive protein, MPO: myeloperoxidase, PSH: thiol proteins, Cu: copper, Zn: zinc.

Association		Correlation	*p*-Value
Cu	ROOH	0.95	<0.001
Cu/Zn	CRP	0.82	0.007
PAOT^®^score	Vitamin E/cholesterol	0.82	0.007
albumin	MPO	−0.75	0.020
Cu	γ-tocopherol	−0.75	0.020
GSH	PSH	0.73	0.026
Cu	Cu/Zn	0.72	0.030
γ-tocopherol	ROOH	−0.63	0.067
PAOT^®^score	Zn	0.63	0.067
CRP	Vitamin E	−0.61	0.081
MPO	PSH	−0.61	0.081
Cu/Zn	ROOH	0.58	0.099

## Data Availability

The datasets analyzed during the current study are available from the corresponding author on reasonable request.

## Supplementary Materials

The following are available online at https://www.mdpi.com/2076-3921/10/2/257/s1, Figure S1: Individual plasma values in antioxidants observed in COVID-19 patients (N = 9). Figure S2: Individual plasma values in trace elements and markers of lipid peroxidation observed in COVID-19 patients (N = 9). Figure S3: Individual plasma or blood values in inflammatory biomarkers observed in COVID-19 patients (N = 9)., Table S1: Statistical comparison of median values for all investigated OS biomarkers between short and long stayers., Table S2: Percentage (%) of patients among short (N = 3) and long stayers (N = 6) having individual values of OS biomarkers below the lower normal value (LNV) and above the upper normal value (UNV).
