# Supplementary material for: Oxidative Stress Status in COVID-19 Patients Hospitalized in Intensive Care Unit for Severe Pneumonia. A Pilot Study
Source: Antioxidants (Basel). 2021 Feb 7;10(2):257. doi: 10.3390/antiox10020257 (PMC7914603; doi:10.3390/antiox10020257)
Supplement: Supplementary file 1 [file antioxidants-10-00257-s001.pdf]

Table S1. Statistical comparison of median values for all investigated OS biomarkers between short and long stayers.

Variable	Reference interval	Median (range)		P-value
		9 (7 - 11) days (N = 3)	41 (38.7 - 43) days (N = 6)	
Antioxidants				
vitamin C (μg/mL)	6.2 - 15.2	2.99 (2.72 - 3.12)	4.97 (3.84 - 7.51)	0.0001
vitamin E as α-tocopherol (μg/mL)	8.6 - 19.2	16.6 (6.25 - 23.1)	19.1 (13.54 - 21.10	0.4
vitamin E/cholesterol (μg/g)	4.4 - 7	10.8 (8.64 - 15.50)	11.1 (9.27 - 12.79)	0.99
γ-tocopherol (μg/mL)	0.39 - 2.42	1.27 (0.55 - 1.29)	0.71 (0.52 - 1.05)	0.71
β-carotene (μg/mL)	0.06 - 0.68	0.10 (0.06 - 0.10)	0.21 (0.12 - 0.29)	0.023
thiol proteins (μM)	314 - 516	249 (211 - 255)	251 (211 - 255)	0.87
glutathione (μM)	717 - 1110	704 (510 - 1090)	598 (531 - 666)	0.38
oxidized glutathione (μM)	0.96 - 10	< 1	< 1	
PAOT®score (U/L)	1.46 - 36.74	9.72 (6.49 - 10.65)	10.53 (6.54 - 11.29)	0.71
glutathione peroxidase (UI/g Hb)	20 - 56	67.5 (55.1 - 76.40)	73.75 (69.6 - 80.4)	0.38
albumin (g/L)	32-46	31 (27-31)	28 (27 - 36)	0.90
Trace elements				
copper (mg/mL)	0.70 - 1.1	0.98 (0.47 - 0.78)	1.22 (0.47 - 1.52)	0.90
zinc (mg/mL)	0.70 - 1.20	0.83 (0.45 - 0.92)	0.91 (0.81 - 1.27)	0.38
selenium (μg/mL)	73 - 110	51 (28 - 67)	97 (74 - 105)	0.023
Biomarkers of lipid peroxidation				
ROOH (μM)	0 - 432	538 (191 - 1127)	676 (138 - 1846)	0.90
ox-LDL (ng/mL)	28 - 70	46 (36 - 69)	55 (35 - 79)	0.76
Ab-ox-LDL	200 - 600	700 (68 - 1200)	253 (42 - 1200)	0.72
Sources of ROS production				
copper/zinc ratio	1 - 1.17	1.55 (0.93 - 2.17)	1.27 (0.54 - 1.64)	0.71
white blood cells ($10^3/mm^3$)	4.60 - 10.10	32.8 (2.1 - 182.7)	35.6 (11.6 - 56.8)	0.99
neutrophils (%)	42 - 71	70 (54 - 87)	76 (74 - 85)	0.71
myeloperoxidase (ng/mL)	27 - 72	115 (88 - 336)	75 (49 - 187)	0.25
C-reactive protein (mg/L)	0 - 5	32.8 (2.1 - 182.7)	35.65 (11.65 - 56.8)	0.99

Table S2: Percentage (%) of patients among short (N = 3) and long stayers (N = 6) having individual values of OS biomarkers below the lower normal value (LNV) and above the upper normal value (UNV).

	% values below LNV	
	short stayers (N = 3)	long stayers (N = 6)
vitamin C	100	66.6
vitamin E (α-tocopherol)	33.3	0
vitamin E (γ-tocopherol)	0	0
β-carotene	0	0
thiol proteins (PSH)	100	100
glutathione (GSH)	66.6	100
PAOT®score	0	0
zinc	33.3	0
selenium	100	0
	% values higher than UNV	
	short stayers (N = 3)	long stayers (N = 6)
glutathione peroxidase (GPx)	66.6	100
copper (Cu)	33.3	66.6
Cu/Zn	66.6	50
lipid peroxides (ROOH)	66.6	66.6
oxidized LDL (ox-LDL)	0	33.3
antibodies against ox-LDL (Ab-ox-LDL)	33.3	50
white blood cells ($10^3/mm^3$)	66.6	16.6
neutrophils	100	50
myeloperoxidase (MPO)	100	33.3
C-reactive protein (CRP)	66.6	66.6

Figure S1. Individual plasma values in antioxidants observed in COVID-19 patients (N = 9). The rectangle in gray represents the reference interval. The black line indicates the median value. LNV: lower normal value; UNV: upper normal value. k: number of COVID-19 values below* or above** the reference interval.

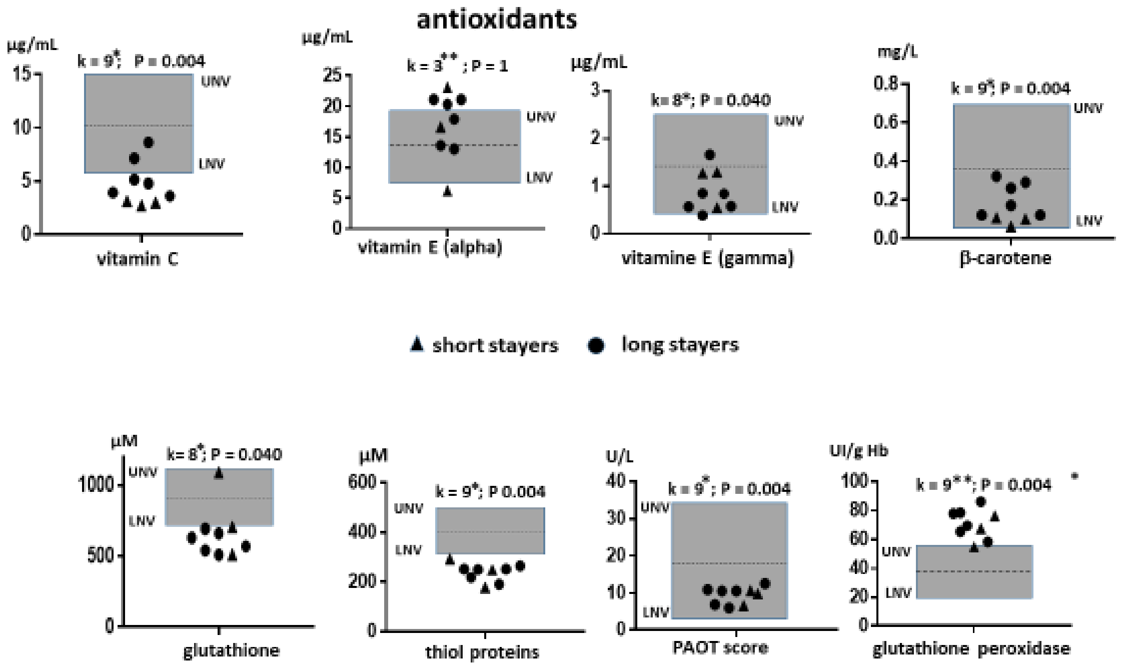

Figure S2. Individual plasma values in trace elements and markers of lipid peroxidation observed in COVID-19 patients (N = 9). The rectangle in gray represents the reference interval. The black line indicates the median. LNV: lower normal value; UNV: upper normal value. k: number of COVID-19 values below* or above** the reference interval.

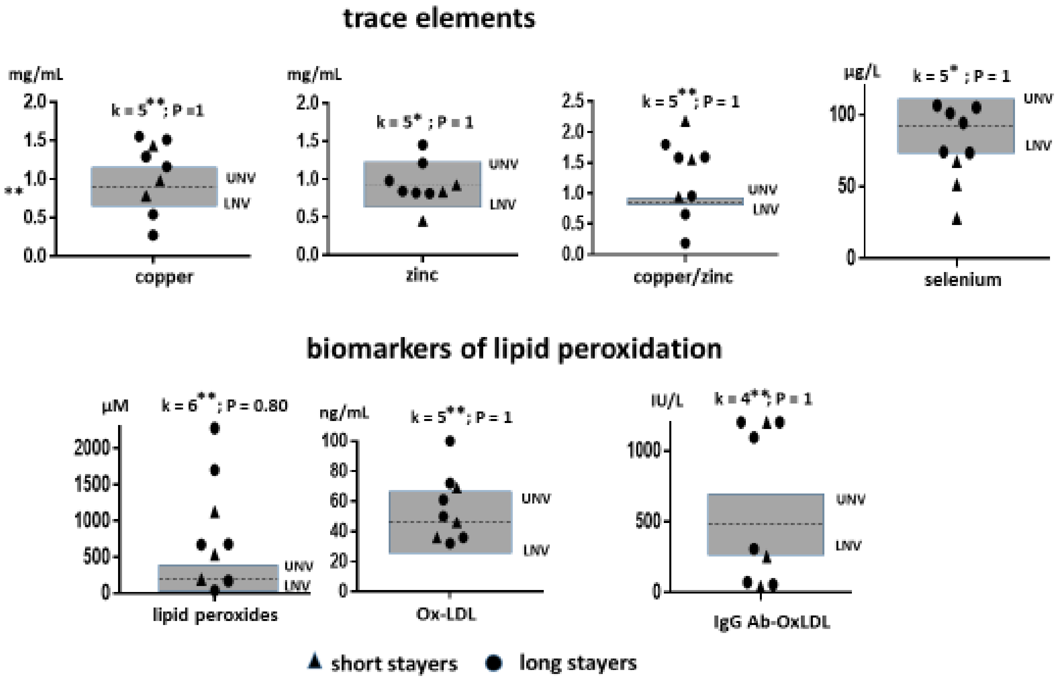

Figure S3. Individual plasma or blood values in inflammatory biomarkers observed in COVID-19 patients (N = 9). The rectangle in gray represents the reference interval. The black line indicates the median with its corresponding value. LNV: lower normal value; UNV: upper normal value. k: number of COVID-19 values below* or above** the reference interval.

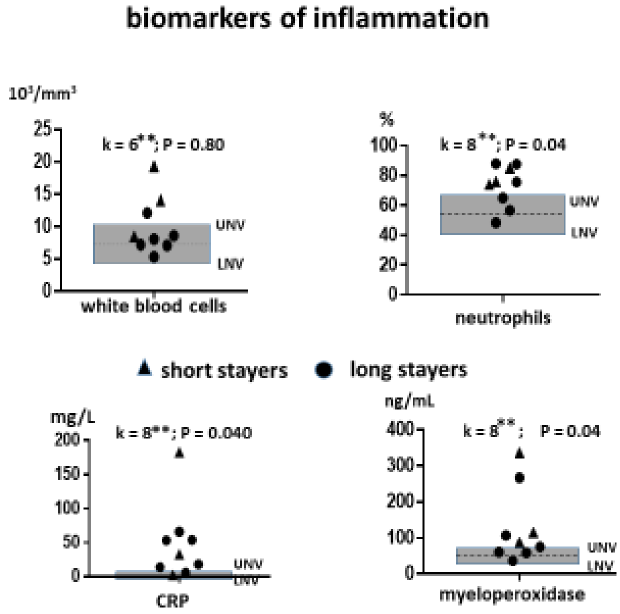

**

*
